# Detection of Type B Aortic Dissection in the Emergency Department with Point-of-Care Ultrasound

**DOI:** 10.5811/cpcem.2019.5.42928

**Published:** 2019-07-08

**Authors:** Emily Earl-Royal, Phi D. Nguyen, Al’ai Alvarez, Laleh Gharahbaghian

**Affiliations:** * Stanford School of Medicine, Department of Emergency Medicine, Palo Alto, California; † Kaiser Permanente Sacramento Medical Center, Department of Emergency Medicine, Sacramento, California

## Abstract

Aortic dissection (AD) is a rare, time-sensitive, and potentially fatal condition that can present with subtle signs requiring timely diagnosis and intervention. Although definitive diagnosis is most accurately made through computed tomography angiography, this can be a time-consuming study and the patient may be unstable, thus preventing the study’s completion. Chest radiography (CXR) signs of AD are classically taught yet have poor diagnostic reliability. Point-of-care ultrasound (POCUS) is increasingly used by emergency physicians for the rapid diagnosis of emergent conditions, with multiple case reports illustrating the sonographic signs of AD. We present a case of Stanford type B AD diagnosed by POCUS in the emergency department in a patient with vague symptoms, normal CXR, and without aorta dilation. A subsequent review of CXR versus sonographic signs of AD is described.

## INTRODUCTION

Aortic dissection (AD) is a rare and potentially fatal condition that can have delayed diagnosis in the emergency department (ED). Atypical signs and symptoms,^1^ patient instability that prevents transport to radiology, and a long turn-around time for computed tomography angiography (CTA) imaging and its results all contribute to diagnostic limitations.^2^ Clinically, AD may present with chest, abdominal or back pain, syncope or near-syncope, weakness, shortness of breath, or a sense of impending doom with pulse deficits, blood pressure differentials, neurologic deficits, or signs of cardiogenic or hypovolemic shock. For every hour delay in AD diagnosis, there is an estimated 1% increased risk in mortality, and AD continues to have a mortality of 25–30%.^2^ The International Registry of Acute Aortic Dissection (IRAD) holds the largest database for AD presentation, imaging, and management. IRAD demonstrates that although clinical features haven’t changed in 20 years, imaging has been optimized, including that of echocardiography.^3^–^5^ Emergency physicians must maintain a high index of suspicion and use available resources, including point-of-care ultrasound (POCUS).

## CASE REPORT

A 49-year-old male with a new diagnosis of hypertension presented to the ED after leaving against medical advice from an outside hospital due to waiting multiple hours for CTA imaging. He reported two days of sudden onset back pain associated with discomfort while swallowing, without blood pressure control at the prior hospital. Initial vital signs included a heart rate of 97 beats per minute, blood pressure of 184/100 millimeters of mercury, respiratory rate of 16 breaths per minute, oxygen saturation of 95% on room air, and temperature of 98.2 degrees Fahrenheit. Physical exam revealed a patient in no acute distress, clear lung sounds, no murmur, and a soft, non-distended, non-tender abdomen with no palpable masses and symmetric distal pulses. Electrocardiogram showed normal sinus rhythm without ischemic changes.

**Image 1:**
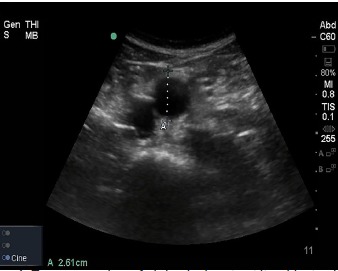
Transverse view of abdominal aorta at its widest point measures 2.61 centimeters.

**Image 2:**
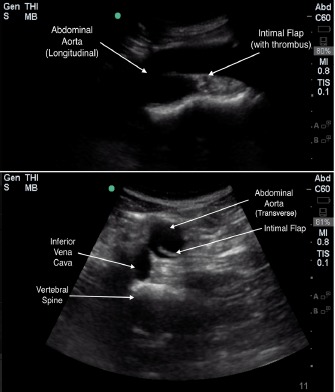
Transverse (bottom) and longitudinal (top) views of abdominal aortic dissection with intimal flap floating in true lumen.

**Image 3:**
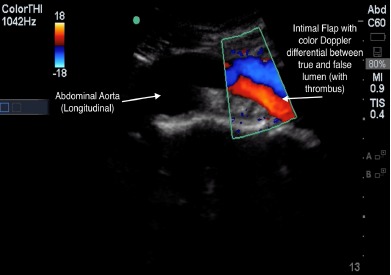
Longitudinal view of abdominal aortic dissection with true lumen, intimal flap, and echogenic false lumen. Superimposed color Doppler demonstrating flow differences in true and false lumen of abdominal aortic dissection.

**Image 4:**
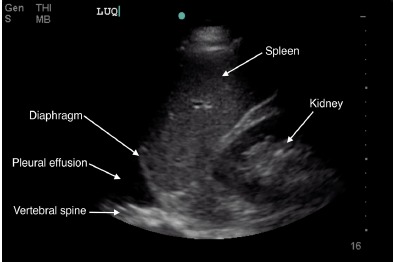
Left coronal view of thoracic cavity showing anechoic (black) fluid above the diaphragm, indicative of pleural effusion.

POCUS of the abdominal aorta and a transthoracic echocardiogram with lung views using a Sonosite Edge 5-2 Megahertz (MHz) curvilinear and 5-1MHz phased array transducer, respectively, showed an abdominal aorta intimal flap with color Doppler differential between the true and false lumen (Images 1-4 and Video), normal cardiac contractility and ascending aorta size without pericardial effusion with poor visualization of the descending aorta, and a left-sided pleural effusion. A rapid diagnosis of AD was made, continuous blood pressure monitoring and control measures were implemented, and the vascular surgery service was consulted within minutes after ED arrival. Chest radiography (CXR) showed no acute cardiopulmonary disease. A stat CTA chest/abdomen/pelvis confirmed a Stanford type B AD originating between the left common carotid and the left subclavian artery extending through the chest and abdomen to the proximal bilateral common iliac arteries. He underwent medical management with stable repeat CTA on hospital day seven and was subsequently discharged.

**CPC-EM Capsule**

What do we already know about this clinical entity?

Aortic dissection (AD) is a potentially fatal clinical emergency with diagnostic limitations. A high clinical suspicion is required.

What makes this presentation of disease reportable?

Point-of-care ultrasound (POCUS) adds to the diagnostic process with both direct and indirect data, and can illustrate the definitive diagnostic sign for the diagnosis of AD.

What is the major learning point?

POCUS can help identify AD with higher accuracy than chest radiograph, as well evaluate for other causes of chest pain.

How might this improve emergency medicine practice?

POCUS can aid the emergency physician in the evaluation of patients suspected of having an AD.

## DISCUSSION

Violation of the intimal layer of the aorta, allowing blood to dissect between the intimal and adventitial layers, defines AD. Further classification of AD includes which portions of the aorta are involved, with Stanford type A involving the ascending aorta and Stanford type B involving the descending aorta.^6^ It is a rare condition with a reported incidence of 2.9 per 100,000 persons per year.^7^ Untreated AD mortality rates approach 25% at 24 hours and 75% by two weeks. Type B dissection treated medically carries a mortality of 10.7% while the mortality rate of those treated surgically being higher at 31.4%, mainly due to aortic rupture and visceral ischemia.^3^

AD is a clinical diagnostic challenge. The presentation can vary from no deficits from organ damage to myocardial infarction and stroke. Approximately 6–15% of patients have no pain; however, they carry an increased mortality compared with painful AD.^7^,^8^ The most common presenting complaint is severe, sharp chest pain and is more common with type A dissection; back and abdominal pain occurs more with type B dissection.^3^,^8^ Hypertension, present in up to 72% of patients with AD, is the most important risk factor and causes increased shear forces that propagate the dissection.^3^ Clinical variability contributes to emergency physicians’ suspicion for AD in confirmed cases being at only 43%.^9^ Our patient presented with new hypertension, back pain, and odynophagia, a rare clinical manifestation of AD discussed in only a few case reports.^10^–^12^

Timely lab and imaging tests for AD evaluation have limitations. Some evidence shows that D-dimer may help when matched with the AD detection risk score,^13^,^14^ while troponin can be an indirect marker for patients with resultant myocardial ischemia. Advanced imaging would still be required.^3^,^14^ As with our patient, the absence of aortic dilation does not exclude AD diagnosis, with a normal CXR occurring in 12.4% of patients with AD and mostly in those who have a normal aorta size.^3^,^15^,^16^ However, pleural effusion on CXR is an independent predictor of mortality in type B dissection.^17^ While there was no pleural effusion seen on our patient’s CXR, we did see a pleural effusion on POCUS.

The largest database of AD patients, IRAD, bases the diagnosis on medical history, imaging study, direct visualization at surgery, or post-mortem examination. It reviews patient presentation, lab testing, imaging studies, and AD management.^3^,^18^ Although CTA is the gold standard for AD diagnosis, emergency physicians order a CXR to screen for other causes while knowing there are specific CXR signs of AD. However, CXR for AD has limitations, including an inability to exclude AD and the absence of the classic CXR sign for AD, mediastinal widening, in 37.4% of patients with AD.^3^,^19^,^20^ With imaging advances, there has been increased utilization of CTA (used as the initial imaging modality in 61% of patients), magnetic resonance imaging (MRI), and echocardiography (transesophageal [TEE] and/or transthoracic [TTE]), used as the initial imaging modality in 33% of patients.^3^,^8^,^20^ CTA and MRI imaging may not be readily available, while TEE requires mobilizing resources and sedation and cannot assess the abdominal aorta. Ultimately, IRAD showa that most patients required more than one imaging study.^3^,^18^

POCUS is readily available, provides rapid evaluation and dynamic imaging for emergency physicians, and lacks the need for contrast or ionizing radiation. It is most critical in patients with undifferentiated shock where a protocol exists that includes an evaluation of the heart, lung, and aorta.^21^ TTE, lung, and transabdominal aorta POCUS views can show the following sonographic findings diagnostic of, or as a consequence of, AD: intimal flap separating the true and false lumens (sensitivity 67–79%; specificity 99–100%); aorta dilation (sensitivity 95%); intra-aorta thrombus; pericardial effusion (sensitivity 96%, specificity 98%); aortic regurgitation; wall motion abnormalities; and left pleural effusion (accuracy 93%) (Table 1).^22^–^28^ TTE has a sensitivity of 77–80% and a specificity of 93–96% for identifying proximal AD, while the sensitivity for distal AD is slightly lower.^20^ Abdominal aorta POCUS assessment for aneurysm has a sensitivity and specificity of over 98%, with abdominal AD assessment described in case reports.^29^–^33^ As seen in our patient, POCUS demonstrates the extent of the dissection, with color Doppler showing differences of blood flow between the true and false lumen, further increasing diagnostic sensitivity.^34^

As opposed to CXR, POCUS is distinctly able to visualize an intimal flap, which is required for the definitive diagnosis of AD. The dynamic nature of POCUS allows for flap assessment in several angles and locations to ensure that flap motion is independent of surrounding structures, is pulsatile, and is contained within the aorta. Characterization of the false lumen of AD by POCUS involves visualization of a wedge-like angle where the flap meets the aortic wall (“beak sign”) and strand-like structures in the lumen (“cobwebs”).^20^ All of the above findings are seen in our patient, with case reports and case series that describe intimal flap visualization (Images 1–4 and Video).^23^–^28^ POCUS applications, including those needed for AD evaluation, are part of the list of ultrasound applications expected for emergency physician training and privileging under the American College of Emergency Physicians.^35^

There are inherent limitations to POCUS, particularly with regard to sonographer skill and experience, sonographic artifacts, and patient-centered challenges such as body habitus, bowel gas, and overall cooperation.^20^,^27^ Also, portions of the thoracic aorta cannot be visualized by POCUS and evaluation of intimal flap extension into smaller arteries is limited. Further investigation is needed to determine the true accuracy of POCUS with Type B AD diagnosis especially when it involves the abdominal aorta. Whether the list of direct and indirect sonographic signs independently or collectively should be used to increase diagnostic sensitivity requires further investigation.

## CONCLUSION

AD is a rare, potentially fatal, and clinically difficult diagnosis. Subtle signs and symptoms, diagnostic challenges and limitations of CXR, and difficulties in obtaining timely definitive diagnosis using CTA, all contribute to diagnostic delays. As described in this case report, POCUS can accurately diagnose AD rapidly when multiple views are obtained that include the heart, lung, and thoracic and abdominal aorta in order to assess for the various sonographic signs for AD.

## Supplementary Information

Video. Transverse view of abdominal aortic dissection with intimal flap.

## Figures and Tables

**Table:** Summary of chest radiography versus point-of-care ultrasound (POCUS) signs of non-traumatic aortic dissection.^3^,^20^,^29^,^32^,^36^–^40^

Imaging sign	Chest radiograph	POCUS (views)
Intimal flap (definitive diagnosis of AD)	No	Yes (PSL [aortic root and ascending aorta], PSL and AP4 [portions of descending aorta], suprasternal aortic arch, abdominal descending aorta) (sensitivity 88–99%)
Intramural hematoma/thrombus (definitive diagnosis of AD)	Yes (sensitivity 49–63%)	Yes (PSL [aortic root and ascending aorta], suprasternal aortic arch, abdominal descending aorta) (sensitivity 88%)
Left-sided pleural effusion	Yes (sensitivity 19%)	Yes (lung) (sensitivity 94%)
Pericardial effusion/tamponade (enlarged cardiac silhouette)	Yes (sensitivity 26%)	Yes (SX, PSL) (sensitivity 96%)
Wall motion abnormality	No	Yes (SX, PSL, AP4)
Thoracic aorta size/contour (wide mediastinum)	Yes (sensitivity 49–67%)	Yes (PSL, suprasternal aortic arch) (sensitivity 93%)
Abdominal aorta size	No	Yes (abdominal aorta) (sensitivity 99–100%)

*AD,* aortic dissection; *PSL,* parasternal long; *AP4,* apical four chamber; *SX,* subxiphoid.
